# Quantum Annealing for Prime Factorization

**DOI:** 10.1038/s41598-018-36058-z

**Published:** 2018-12-05

**Authors:** Shuxian Jiang, Keith A. Britt, Alexander J. McCaskey, Travis S. Humble, Sabre Kais

**Affiliations:** 1 Department of Computer Science, Purdue University, West Lafayette, IN 47906 USA; 2 Quantum Computing Institute, Oak Ridge National Laboratory, Oak Ridge, TN 37831 USA; 3 Department of Chemistry, Physics and Birck Nanotechnology Center, Purdue University, West Lafayette, IN 47906 USA

## Abstract

We have developed a framework to convert an arbitrary integer factorization problem to an executable Ising model by first writing it as an optimization function then transforming the k-bit coupling (*k* ≥ 3) terms to quadratic terms using ancillary variables. Our resource-efficient method uses ${\mathscr{O}}({\mathrm{log}}^{2}(N))$ binary variables (qubits) for finding the factors of an integer *N*. We present how to factorize 15, 143, 59989, and 376289 using 4, 12, 59, and 94 logical qubits, respectively. This method was tested using the D-Wave 2000Q for finding an embedding and determining the prime factors for a given composite number. The method is general and could be used to factor larger integers as the number of available qubits increases, or combined with other ad hoc methods to achieve better performances for specific numbers.

## Introduction

Integer factorization reduces an integer *N* to its factors *p* and *q* such that *pq* = *N*. While this fundamental problem in number theory is computationally hard in practice, integer factorization is not believed to belong to the class of NP-hard problems. However, all known classical factoring algorithms which are deterministic and don’t have unproven hypotheses require time exponential in log*N*. For example, the fastest, known classical algorithm for integer factorization is the general number field sieve method^1^, which scales exponentially in the number of operations required with respect to the integer *N*. Thus, the integer factorization problem has been used as a basic hardness assumption for many encryption methods including the widely deployed RSA cryptographic system. With broad applications in cryptographic data storage and communications^2^, identifying new methods for integer factorization plays an important role in modern information security.

Quantum computing theory has the potential to reduce the number of operations required for solving the integer factorization problem. Within the circuit model of quantum computation, Shor’s algorithm is perhaps the most well-known method for integer factorization, in which the number of operations to factorize an integer *N* is polynomial in the size log*N*^3^. This exponential speedup over the general number field sieve is achieved by reducing factorization to the order-finding problem. Several experimental demonstrations using quantum computing hardware have validated the correctness of Shor’s algorithm for small integer values, including early work by Vandersypen *et al*.^4^ to factorize *N* = 15 using seven spin-1/2 nuclei in a molecule as qubits. Subsequent experiments by Lanyon *et al*.^5^, Lu *et al*.^6^, and Politi *et al*.^7^ implemented compiled versions of Shor’s algorithm using photonic systems for factoring 15. Martín-López *et al*.^8^ factored 21 using qubit recycling, and Lucero *et al*.^9^ used superconducting qubits to factor 15. Geller *et al*.^10^ used a simplified version of Shor’s algorithm for factoring products of the Fermat primes 3, 5, 17, 257, and 65537. Recent work from Grosshans *et al*. has shown how factoring safe semi-primes using the quantum order-finding algorithm can reduce the failure probability^11^.

An equally powerful model of quantum computing is the adiabatic quantum computing (AQC) model^12,13^, which can also solve the integer factorization problem. Peng *et al*. first developed integer factorization within AQC by reducing it to unconstrained optimization and solving this problem using adiabatic quantum dynamics. They further validated these ideas experimentally using a three-qubit NMR quantum processor for the case of *N* = 21^14^, while Xu *et al*. subsequently factored 143 using similar NMR technology^15^. Schaller *et al*. developed a novel approach based on multiplication tables that can be cast as an optimization, which they have demonstrated for biprimes up to *N* = 217^16^. Dridi *et al*. furthered these ideas by using Gröbner bases to reduce the number of auxiliary variables required and simplify equations, thereby enabling a demonstration of factorization up to 223357^17^.

In this contribution, we introduce a new procedure for solving the integer factorization problem using quantum annealing^18,19^ which utilizes adiabatic quantum computation. Differ from the recent work^20^ which sketched the hardware design of reversible multiplier to achieve factorization, we provide specific mathematical derivations to be tested on the existing hardware. We begin by describing a direct method for integer factorization that reduces the problem to unconstrained optimization. We review how this optimization problem can be reduced to a quadratic form Ising Hamiltonian and be solved using quantum annealing. We then describe a modified multiplication table method that reduces the overall resource requirements on the optimization problem and permits methods to account for constraints that may appear in quantum annealing hardware, such as connectivity and number of qubits. Our modification also reduces the range of coefficients in the underlying cost function without increasing the number of qubits required. This method avoided the time-consuming preprocessing steps^17^ to achieve comparable results, and could be combined with other ad-hoc function simplification methods to further reduce the number of qubits or other aspects. We finally tested both methods with results from experimental demonstrations using quantum annealing hardware.

## Background

Quantum Annealing was introduced^18^ to solve optimization problems using quantum fluctuations to transit to the ground state, compared to simulated annealing which uses thermal fluctuations to get to the global minimum. Quantum fluctuations such as quantum tunneling^21^ provide ways of transitions between states. The transverse field controls the rate of the transition, as the role of temperature played in simulated annealing.

Farhi *et al*.^12^ remodeled the procedure as Adiabatic Quantum Computing(AQC), which finds the energetic ground state of a problem Hamiltonian by adiabatically evolving the quantum state. If the system begins at the ground state, after the adiabatic evolution, the system will remain at the ground state. By combining the initial Hamiltonian *H*_*B*_ and the problem Hamiltonian *H*_*P*_ linearly, the system could be defined as a time-dependent Hamiltonian $H(t)=(1-\frac{t}{T}){H}_{B}+\frac{t}{T}{H}_{P}$, where the duration *T* defines the time-scale for evolution and controls the rate at which the time-dependent Hamiltonian changes, the initial Hamiltonian *H*_*B*_ is of the form ${H}_{B}=-\,{\sum }_{i=1}^{L}\,{\sigma }_{x}^{(i)}$ over *L* qubits with the Pauli operator ${\sigma }_{x}^{(i)}$ defining the *x*-basis of the *i*-th qubit. The problem Hamiltonian *H*_*P*_ is in the form of Ising model over *L* qubits as

1 $${H}_{P}=\sum _{i=1}^{L}\,{h}_{i}{\sigma }_{i}^{z}+\sum _{i < j}^{L}\,{J}_{ij}{\sigma }_{i}^{z}{\sigma }_{j}^{z}$$

where ${\sigma }_{z}^{(i)}$ defines the *z*-basis for the *i*-th qubit and the local fields *h*_*i*_ and couplings *J*_*ij*_ define the problem instance.

Computation within the AQC model evolves the *L*-qubit quantum state under the time-dependent Hamiltonian *H*(*t*) according to the Schrödinger equation $i\hslash \frac{\partial }{\partial t}|\psi (t)\rangle =H(t)|\psi (t)\rangle$, where |*ψ*(*t*)〉 is the state of the system at time *t* ∈ [0, *T*] and we will set ℏ = 1. Let |*ϕ*_*i*_(*t*)〉 be the *i*-th instantaneous eigenstate, such that *H*(*t*)|*ϕ*_*i*_(*t*)〉 = *E*_*i*_(*t*) |*ϕ*_*i*_(*t*)〉, and let the initial state of the system be the ground state at time *t* = 0, such that |*ψ*(0)〉 = |*ϕ*_0_(0)〉. According to the adiabatic theorem^22^, the system state will remain in the instantaneous ground state of the time-dependent Hamiltonian provided the evolution is sufficiently slow to prevent excitations to higher-lying states. Under these idealized adiabatic conditions, the system will evolve into the energetic ground state of the problem Hamiltonian as |*ψ*(*T*)〉 = |*ϕ*_0_(*T*)〉. This prepared quantum state of *L* qubits is then measured to generate a classical string of *L* bits that represents the solution to the encoded factorization problem.

Several practical considerations limit the applicability of the AQC model for solving optimization problems. Foremost is the requirement that changes in the quantum state must be adiabatic, i.e, slow, relative to the internal timescales of the instantaneous Hamiltonian *H*(*t*). Theoretical analyses of this requirement provide a best lower bound on the time *T* as *O*(Δ^−2^), where Δ is the minimum energy gap within the instantaneous eigen spectrum of *H*(*t*)^13^. However, the minimum spectral gap is dependent on the specific instances of the initial and final Hamiltonians and the interpolation between them. A priori knowledge of spectral gap information would provide a significant insight into the underlying optimization problem, if not the solution directly, and therefore is an impractical expectation for a computational method. In addition, the pure-state model for AQC fails to account for finite-temperature effects observed in actual hardware as well as unexpected environmental coupling, unpredictable control noise, unwanted crosstalk, and other imperfections.

Practically quantum annealing relaxes the AQC with guarantee that the observed final state corresponds to the energetic ground state. However, the probability to observe the energetic ground state may be reduced due to physical noise and non-adiabatic dynamics. The resulting error rate *p*_*e*_(*L*) characterizes the quantum annealing dynamics, which is most accurately modeled as an open quantum system of *L*-qubits evolving the presence of an uncontrolled environment. The quantum annealing model is therefore more robust to the above practical considerations but it is necessarily a probabilistic computational model. Statistical sampling of a quantum annealing computation is always necessary to gather confidence in the observed result. Quantum annealing may also be interpreted as a meta-heuristic for managing noisy AQC computation, whereby the aggregate likelihood of success *p*_*s*_ is determined by the number of samples *S* as *p*_*s*_ = 1−(*p*_*e*_)^*S*^. The number of samples necessary to achieve a desired probability of success is therefore *S* ≥ log(1−*p*_*s*_)/log(*p*_*e*_). For a fixed annealing duration *T* and probability of success *p*_*s*_, we may expect the probability of error *p* to increase as the size of the system increases, i.e., as *L* increases. The rate at which the sample number *S* increases with system size plays an important role in determining the computational complexity of using the quantum annealing model. For example, an error rate that increases exponentially with system size, i.e., *p*_*e *_∝ exp(*L*), yields a sampling rate that increases linearly.

A related practical consideration is the resource efficiency with which quantum annealing can be implemented. Specifically, the number of qubits necessary to realize the problem Hamiltonian *H*_*P*_ influences not only the number of samples required but also the feasibility of demonstrating the method on available hardware. The most general case of *L*-qubit Hamiltonian may include all-to-all connectivity, whereby each qubit must interact with every other qubits. However, most of the existing hardware does not permit such connectivity directly, and methods for realizing implicit connections have been developed^23,24^. In our implementation of integer factorization using the Ising Hamiltonian, it is necessary to compose a problem Hamiltonian in terms of pairwise interactions, and we develop an efficient transformation of the factoring problem Hamiltonian into pair-wise coupling.

## Methods

We describe two methods for implementing integer factorization within the quantum annealing model. We found these two corresponding Hamiltonians *H*_*P*_ to encode the factors of an input integer *N*, such that the energetic ground state corresponds to factorization of the input. The first is a direct method to compute the factors of *N* = *pq* by constructing the associated optimization problem as an Ising Hamiltonian. The second method is based on the modified multiplication tables to translate the problem into the Ising Hamiltonian. We test our methods using experimental quantum computing hardware appropriate for quantum annealing. The D-Wave 2000Q processor natively implements an Ising model Hamiltonian and provides programmable control over the parameters *h*_*i*_ and *J*_*i*,*j*_ as well as the annealing duration *T*.

### Direct Method

Our direct method factors *N* = *pq*, where *p* and *q* are prime numbers. We set ${l}_{1}=\lfloor {\mathrm{log}}_{2}(p)\rfloor$ and ${l}_{2}=\lfloor {log}_{2}(q)\rfloor$. Because *p* and *q* are prime numbers, we use the binary representations $p={({x}_{{l}_{1}-1}{x}_{{l}_{1}-2}\mathrm{...}{x}_{1}\mathrm{1)}}_{2}$ and $q={({x}_{{l}_{1}+{l}_{2}-2}{x}_{{l}_{1}+{l}_{2}-3}\mathrm{...}{x}_{{l}_{1}}\mathrm{1)}}_{2}$, where *l*_1_ > *l*_2_ and *x*_*i*_ ∈ {0, 1} for *i* = 1 to *l*_1_ + *l*_2_−2. We define the cost function $f({x}_{1},\,{x}_{2},\,{x}_{3},\,{x}_{4},\,\mathrm{...,}\,{x}_{{l}_{1}+{l}_{2}-2})={(N-pq)}^{2}$, and explicit multiplication of the binary representations for *p* and *q* yields a sum of binary products. We reduce the resulting 3-local terms to 2-local terms using the following equivalence^24^: for *x*, *y*, *z* ∈ {0, 1}, *xy* = *z* iff *xy*−2*xz*−2*yz* + 3*z* = 0, and *xy* ≠ *z* iff *xy*−2*xz*−2*yz* + 3*z* > 0. Therefore,

$${x}_{1}{x}_{2}{x}_{3}={x}_{4}{x}_{3}+\mathrm{2(}{x}_{1}{x}_{2}-2{x}_{1}{x}_{4}-2{x}_{2}{x}_{4}+3{x}_{4})\,{\rm{if}}\,{x}_{4}={x}_{1}{x}_{2}$$

and

$${x}_{1}{x}_{2}{x}_{3} < {x}_{4}{x}_{3}+\mathrm{2(}{x}_{1}{x}_{2}-2{x}_{1}{x}_{4}-2{x}_{2}{x}_{4}+3{x}_{4})\,{\rm{if}}\,{x}_{4}\ne {x}_{1}{x}_{2}$$

thus, the *x*_1_*x*_2_*x*_3_ term may be transformed to quadratic form by replacing *x*_1_*x*_2_ with *x*_4_ plus a constraint in the form of a penalty term:

2 $${\rm{\min }}({x}_{1}{x}_{2}{x}_{3})=\,{\rm{\min }}({x}_{4}{x}_{3}+\mathrm{2(}{x}_{1}{x}_{2}-2{x}_{1}{x}_{4}-2{x}_{2}{x}_{4}+3{x}_{4})$$

By introducing a new variable and adding the penalty term, we are able to transform 3-local terms to 2-local terms.

For integer factorization, we require $$(\begin{array}{c}{l}_{1}\\ 2\end{array})+(\begin{array}{c}{l}_{2}\\ 2\end{array})=\frac{{l}_{1}({l}_{1}-\mathrm{1)}}{2}+\frac{{l}_{2}({l}_{2}-\mathrm{1)}}{2}$$ auxiliary variables to form a quadratic cost function, and when *l*_1_ = *l*_2_ = *l* this number is *l* × (*l*−1). Counting the variables to denote the factors themselves, the quadratic function requires *L* = 2 × (*l*−1) + *l* × (*l*−1) = (*l* + 2) × (*l*−1) binary variables in total. Since $l={\mathscr{O}}(\mathrm{log}(N))$, $L={\mathscr{O}}({\mathrm{log}}^{2}(N))$. We could also let $p={\mathrm{(1}{x}_{{l}_{1}-2}\mathrm{...}{x}_{1}\mathrm{1)}}_{2},\,q={\mathrm{(1}{x}_{{l}_{1}+{l}_{2}-4}\mathrm{...}{x}_{{l}_{1}-1}\mathrm{1)}}_{2}$ when lengths of *p* and *q* are prefixed.

We illustrate this direct method of factorization for the case of *N* = 15. Because ${{\rm{l}}{\rm{o}}{\rm{g}}}_{2}(p)\,\le \,2\, < \,{{\rm{l}}{\rm{o}}{\rm{g}}}_{2}(q)\, < \,4$, we define *p* = (*x*_1_1)_2_ and *q* = (*x*_2_*x*_3_1)_2_. The objective function *f*(*x*_1_, *x*_2_, *x*_3_) = (*N*−*pq*)^2^ may then be reduced to the 3-local form:

$$f(x)=128{x}_{1}{x}_{2}{x}_{3}-56{x}_{1}{x}_{2}-48{x}_{1}{x}_{3}+16{x}_{2}{x}_{3}-52{x}_{1}-52{x}_{2}-96{x}_{3}+196.$$

We reduce the 3-local terms to 2-local terms using the method described above to obtain

$$\begin{array}{rcl}f^{\prime} (x) & = & 200{x}_{1}{x}_{2}-48{x}_{1}{x}_{3}-512{x}_{1}{x}_{4}+16{x}_{2}{x}_{3}-512{x}_{2}{x}_{4}+128{x}_{3}{x}_{4}\\  &  & -52{x}_{1}-52{x}_{2}-96{x}_{3}+768{x}_{4}+\mathrm{196,}\end{array}$$

where

$$\mathop{{\rm{\min }}}\limits_{{x}_{1}{x}_{2}={x}_{4}}f({x}_{1},\,{x}_{2},\,{x}_{3},\,{x}_{4})=\,{\rm{\min }}\,f^{\prime} ({x}_{1},\,{x}_{2},\,{x}_{3},\,{x}_{4})$$

This result is a quadratic unconstrained binary optimization (QUBO) problem over *L* = 4 variables that may be transformed into an equivalent Ising Hamiltonian as defined in Eq. 1 by identifying the binary variable *x*_*i*_ with the *i*-th spin state *s*_*i*_ = 2*x*_*i*_ − 1. For *N* = 15, the local fields *h*^*T*^ and couplings *J* of the Ising problem Hamiltonian are then determined to be

3 $${h}^{T}=(58,\,50,\,12,\,-80)$$

and

4 $$J=(\begin{array}{ccc}25 & -6 & -64\\  & 2 & -64\\  &  & 16\end{array})$$

It is notable that the *L* × *L* coupling matrix J is generally dense on the upper triangle, indicating that *L*(*L*−1)/2 couplings are necessary. Similarly Ising parameters may be generated for other integers *N* by the appropriate quadritization into a QUBO problem and then reduced to Ising form.

### Modified Multiplication Table Method

The second method is based on modified multiplication table. It reduces the range of Ising parameter values used as coefficients for the local fields and couplings. At the meantime, it considers to use a smaller number of carry variables without complicated preprocessing.

The modified multiplication table method uses local minimizations over the products of individual binary substring bits representing the integers *p* and *q*. It divides the multiplication table into several blocks, and considers each block individually. We could also choose the size of each block to get the desired range of parameters and the number of variables, or make a balance between them. A detailed analysis of the range of coefficients is shown in the last section of the supplemental material. Note that the modified multiplication table method does not eliminate the need for quadratization of 4-body and 3-body product terms and auxiliary variables are required. However, this approach does reduce the number of these higher-order terms compared to the direct method, which makes it possible to embed larger problem sizes on currently available quantum hardware.

We describe this method by an illustrative example of *N* = 143, for which *p* = 13 and *q* = 11. Past approaches for integer factorization constructed a system of equations from each column or part of each column in the multiplication table^17,25,26^, which accounts for a carry bit or several carry bits for each part. In our approach, we divide the multiplication table into blocks so that it is only needed to use carries between blocks. This greatly reduced the number of carries, thus the total number of variables reduced too.

As shown in Table 1 for *N* = 143, we introduce two sets of carry bits. We denote them using *c*_*i*_ ∈ {0, 1}, and the two-bit numbers (*c*_2_*c*_1_)_2_ = *c*_2_ × 2 + *c*_1_ and (*c*_4_*c*_3_)_2_ = *c*_4_ × 2 + *c*_3_ represent the carry bits for each of the divided columns in the table. Note that the columns are composed along two-bit domains, so that addition within each block is over two-bit numbers. But the sums are over four-bit numbers. The resulting block system of equations derived from Table 1 is

5 $$\begin{array}{rcl}({p}_{2}+{p}_{1}{q}_{1}+{q}_{2})\times 2+({p}_{1}+{q}_{1}) & = & {c}_{2}\times {2}^{3}+{c}_{1}\times {2}^{2}+{\mathrm{(11)}}_{2}\\  & = & {c}_{2}\times 8+{c}_{1}\times 4+3\\ ({q}_{1}+{p}_{2}{q}_{2}+{p}_{1}+{c}_{2})\times 2+\mathrm{(1}+{p}_{2}{q}_{1}+{p}_{1}{q}_{2}+1+{c}_{1}) & = & {c}_{4}\times {2}^{3}+{c}_{3}\times {2}^{2}+{\mathrm{(01)}}_{2}\\  & = & {c}_{4}\times 8+{c}_{3}\times 4+1\\ \mathrm{(1}+{c}_{4})\times 2+({q}_{2}+{p}_{2}+{c}_{3}) & = & {\mathrm{(100)}}_{2}\\  & = & 4\end{array}$$

**Table 1:** Multiplication table for 13 × 11 or 11 × 13 = 143 in binary.

	2^7^	2^6^	2^5^	2^4^	2^3^	2^2^	2^1^	2^0^
*p*					1	*p*_2_	*p*_1_	1
*q*					1	*q*_2_	*q*_1_	1
					1	*p*_2_	*p*_1_	1
				*q*_1_	*p*_2_ *q*_1_	*p*_1_ *q*_1_	*q*_1_	
			*q*_2_	*p*_2_ *q*_2_	*p*_1_ *q*_2_	*q*_2_		
		1	*p*_2_	*p*_1_	1			
carries		*c*_4_	*c*_3_	*c*_2_	*c*_1_			
*p* × *q* = 143	1	0	0	0	1	1	1	1

Because this modified multiplication table method calculates carries only for each block, it avoids requiring carry bits for each column in the multiplication table. This reduces the overall complexity of the computation by reducing the number of carry bits as well as the number of couplings between bits. In the limit of a single column per block, the conventional multiplication table is recovered, while in the limit of a single equation the direct method is recovered. Instead of making the sum of each column equal to every each bit of the number to be factored as in a conventional multiplication table, we make each block of the multiplication table equal to the corresponding block of the number to be factored. As shown in the appendix material, the equations for these blocks may be reduced to the non-negative cost function

$$\begin{array}{rcl}f(p,\,q,\,c) & = & {\mathrm{(2}{p}_{2}+2{p}_{1}{q}_{1}+2{q}_{2}-8{c}_{2}-4{c}_{1}+{p}_{1}+{q}_{1}-\mathrm{3)}}^{2}\\  &  & +\mathrm{(2}{q}_{1}+2{p}_{2}{q}_{2}+2{p}_{1}+2{c}_{2}-8{c}_{4}-4{c}_{3}+{p}_{2}{q}_{1}+{p}_{1}{q}_{2}\\  &  & +{c}_{1}+{\mathrm{1)}}^{2}+{({q}_{2}+{p}_{2}+{c}_{3}+2{c}_{4}-\mathrm{2)}}^{2}.\end{array}$$

This form may be expanded and further simplified using the property ${x}_{i}^{2}={x}_{i}\,{\rm{for}}\,{x}_{i}=\mathrm{0,}\,1$, while the remaining cubic and higher-order terms like *c*_1_*p*_1_*q*_1_ and *p*_1_*p*_2_*q*_1_*q*_2_ can be reduced to quadratic form by introducing auxiliary variables. In particular, we note that the quadratization of the negative term is similar to the position term, e.g.,

$$\{\begin{array}{lll}-{x}_{1}{x}_{2}{x}_{3}=-{x}_{4}{x}_{3}+\mathrm{2(}{x}_{1}{x}_{2}-2{x}_{1}{x}_{4}-2{x}_{2}{x}_{4}+3{x}_{4}) & {\rm{if}} & {x}_{4}={x}_{1}{x}_{2}\\ -{x}_{1}{x}_{2}{x}_{3} < -{x}_{4}{x}_{3}+\mathrm{2(}{x}_{1}{x}_{2}-2{x}_{1}{x}_{4}-2{x}_{2}{x}_{4}+3{x}_{4}) & {\rm{if}} & {x}_{4}\ne {x}_{1}{x}_{2}\end{array}$$

as detailed in the appendix material, the conversion to QUBO form leads to the parameters for the Ising Hamiltonian. For *N* = 143, this yields the local fields

6 $${h}^{T}=(130.5,\,107.5,\,130.5,\,107.5,\,-41,\,-82,\,3,\,6,\,-137,\,-81,\,-107,\,-81)$$

and the upper triangular coupling matrix

7 $$J=(\begin{array}{rrrrrrrrrrrr} & 2 & 79 & 47.5 & -2 & -4 & -8 & -16 & -148 & -84 & 0 & 0\\  &  & 47.5 & 71 & -8 & -16 & 1 & 2 & 6 & 6 & -124 & -84\\  &  &  & 2 & -2 & -4 & -8 & -16 & -148 & 0 & 0 & -84\\  &  &  &  & -8 & -16 & 1 & 2 & 6 & -84 & -124 & 6\\  &  &  &  &  & 34 & -4 & -8 & -8 & 1 & 2 & 1\\  &  &  &  &  &  & -8 & -16 & -16 & 2 & 4 & 2\\  &  &  &  &  &  &  & 34 & 0 & -4 & -8 & -4\\  &  &  &  &  &  &  &  & 0 & -8 & -16 & -8\\  &  &  &  &  &  &  &  &  & 0 & 1 & 0\\  &  &  &  &  &  &  &  &  &  & 0 & 0\\  &  &  &  &  &  &  &  &  &  &  & 0\end{array})$$

Our approach requires a decision to partition the columns of the multiplication table into blocks, and this choice must balance the number of unknown variables (carries) against the range of coefficients in the problem Hamiltonian. We illustrate this choice for the factorization of biprimes 59989 and 376289 in Tables 2 and 3. Our approach is to set the bit-length of the carry variable for each block based on the largest possible number of that block(the right neighboring columns). For example, the maximum carry for the right-most block in the multiplication table of *N* = 59989 is 3 which requires two bits to represent. Thus, the bit-length of the carry variable for this block is 2, i.e., (*c*_2_*c*_1_)_2_. Similarly, for *N* = 376289, the bit-length of the carry variables for the right-most block is 3, while the bit-length of the carry variable for the third block is 4. Because this bit-length is larger than the size of the fourth block, which has a bit-length of 3, the most significant bit of the carry is included in the neighboring block, i.e., the fifth block in this example.

**Table 2:** Multiplication table for 251 × 239 = 59989 in binary.

	2^15^	2^14^	2^13^	2^12^	2^11^	2^10^	2^9^	2^8^	2^7^	2^6^	2^5^	2^4^	2^3^	2^2^	2^1^	2^0^
p									1	p_6_	p_5_	p_4_	p_3_	p_2_	p_1_	1
q									1	q_6_	q_5_	q_4_	q_3_	q_2_	q_1_	1
									1	p_6_	p_5_	p_4_	p_3_	p_2_	p_1_	1
								q_1_	p_6_q_1_	p_5_q_1_	p_4_q_1_	p_3_q_1_	p_2_q_1_	p_1_q_1_	q_1_	
							q_2_	p_6_q_2_	p_5_q_2_	p_4_q_2_	p_3_q_2_	p_2_q_2_	p_1_q_2_	q_2_		
						q_3_	p_6_q_3_	p_5_q_3_	p_4_q_3_	p_3_q_3_	p_2_q_3_	p_1_q_3_	q_3_			
					q_4_	p_6_q_4_	p_5_q_4_	p_4_q_4_	p_3_q_4_	p_2_q_4_	p_1_q_4_	q_4_				
				q_5_	p_6_q_5_	p_5_q_5_	p_4_q_5_	p_3_q_5_	p_2_q_5_	p_1_q_5_	q_5_					
			q_6_	p_6_q_6_	p_5_q_6_	p_4_q_6_	p_3_q_6_	p_2_q_6_	p_1_q_6_	q_6_						
		1	p_6_	p_5_	p_4_	p_3_	p_2_	p_1_	1							
	c_11_	c_10_	c_9_	c_8_	c_7_	c_6_	c_5_	c_4_	c_3_		c_2_	c_1_				
	1	1	1	0	1	0	1	0	0	1	0	1	0	1	0	1

**Table 3:** Multiplication table for 659 × 571 = 376289 in binary.

2^18^	2^17^	2^16^	2^15^	2^14^	2^13^	2^12^	2^11^	2^10^	2^9^	2^8^	2^7^	2^6^	2^5^	2^4^	2^3^	2^2^	2^1^	2^0^
									1	p_8_	p_7_	p_6_	p_5_	p_4_	p_3_	p_2_	p_1_	1
									1	q_8_	q_7_	q_6_	q_5_	q_4_	q_3_	q_2_	q_1_	1
									1	p_8_	p_7_	p_6_	p_5_	p_4_	p_3_	p_2_	p_1_	1
								q_1_	p_8_q_1_	p_7_q_1_	p_6_q_1_	p_5_q_1_	p_4_q_1_	p_3_q_1_	p_2_q_1_	p_1_q_1_	q_1_	
							q_2_	p_8_q_2_	p_7_q_2_	p_6_q_2_	p_5_q_2_	p_4_q_2_	p_3_q_2_	p_2_q_2_	p_1_q_2_	q_2_		
						q_3_	p_8_q_3_	p_7_q_3_	p_6_q_3_	p_5_q_3_	p_4_q_3_	p_3_q_3_	p_2_q_3_	p_1_q_3_	q_3_			
					q_4_	p_8_q_4_	p_7_q_4_	p_6_q_4_	p_5_q_4_	p_4_q_4_	p_3_q_4_	p_2_q_4_	p_1_q_4_	q_4_				
				q_5_	p_8_q_5_	p_7_q_5_	p_6_q_5_	p_5_q_5_	p_4_q_5_	p_3_q_5_	p_2_q_5_	p_1_q_5_	q_5_					
			q_6_	p_8_q_6_	p_7_q_6_	p_6_q_6_	p_5_q_6_	p_4_q_6_	p_3_q_6_	p_2_q_6_	p_1_q_6_	q_6_						
		q_7_	p_8_q_7_	p_7_q_7_	p_6_q_7_	p_5_q_7_	p_4_q_7_	p_3_q_7_	p_2_q_7_	p_1_q_7_	q_7_							
	q_8_	p_8_q_8_	p_7_q_8_	p_6_q_8_	p_5_q_8_	p_4_q_8_	p_3_q_8_	p_2_q_8_	p_1_q_8_	q_8_								
1	p_8_	p_7_	p_6_	p_5_	p_4_	p_3_	p_2_	p_1_	1									
	c_14_			c_10_	c_9_	c_8_	c_7_	c_6_	c_5_	c_4_	c_3_	c_2_	c_1_					
		c_13_	c_12_	c_11_														
1	0	1	1	0	1	1	1	1	0	1	1	1	1	0	0	0	0	1

We estimate the number of variables needed to construct the Ising Hamiltonian that encodes the factorization problem for *N* = 59989 and 376289. This requires quadratization of the resulting systems of factoring equations followed by reduction to the Ising form, exactly as discussed explicitly above. For the case of *N* = 59989, we have *l*_1_ = *l*_2_ = 6, and therefore 12 variables are required to represent the factors themselves plus 11 variables to denote the carries, while 36 auxiliary variables are required for quadratization of the factoring equations. The total number variables is 59. For *N* = 376289, we have *l*_1_ = *l*_2_ = 8 with 14 carries and 64 auxiliary variables. As noted above, sometimes vary bits in the multiplication table will overlap, as is the case for column 2^14^ of 376289 shown in Table 3. In such circumstances, we just simply add these carries in the table and then use the same method as before to find the corresponding Ising Hamiltonian. Thus, this problem Hamiltonian requires 94 qubits.

Generally, for factoring a biprime number, we use approximately log(*N*) binary variables to encode the integer factors and about log(*N*) binary variables to denote the carries where *N* is the number to be factored. An additional log^2^(*N*)/4 auxiliary binary variables are required for quadratization. Therefore, a total of approximately log^2^(*N*)/4 binary variables are required to represent the problem Hamiltonian and, consequently, a similar number of qubits must be available within hardware. As a point of reference, applying this method to the current factoring record for RSA-768 would require approximately 147456 qubits.

## Experiments

We tested both the direct and modified multiplication table methods using quantum annealing hardware from D-Wave Systems. The D-Wave System hardware consists of a programmable platform of integrated superconducting flux qubits designed to operate within the quantum annealing model. In particular, the hardware system accepts as input a problem Hamiltonian *H*_*P*_ presented in Ising form and the parameters *h* and *J*. The hardware also enables interpolation from the starting Hamiltonian *H*_*B*_ and the final Hamiltonian at a rate controlled by the annealing duration *T*. Measurements of the resulting quantum state are performed in *σ*_*z*_ basis for each qubits.

While the latest 2000Q system contains up to 2048 qubits arranged in a connectivity pattern expressed as a 16-by-16 Chimera graph, this sparse connectivity pattern requires additional resources to ensure the required interactions between the logical variables defining the problem Hamiltonian. This is accomplished by embedding the problem Hamiltonian into the hardware graph while maintaining the logical form of the cost function^27–29^. From the coupling matrix *J*, we define a graph *G* that represents the variables as vertices and non-zero coupling as edges. A minor embedding of *G*(*V*, *E*) into a hardware graph *G*′(*V*′, *E*′) is defined by a mapping $\varphi :G\mapsto G^{\prime}$ such that each vertex *v* ∈ *G* is mapped to a connected subtree *T*_*v*_ of *G*′ and if (*u*, *v*) ∈ *E* then there exist *i*_*u*_, *i*_*v*_ ∈ *G*′ such that *i*_*u*_ ∈ *T*_*u*_, *i*_*v*_ ∈ *T*_*v*_ and (*i*_*u*_, *i*_*v*_) ∈ *E*′. If such a mapping *ϕ* exists between *G* and *G*′, we say *G* is a *minor* of *G*′ and we use *G* ≤ _*m*_*G*′ to denote such relationship.

The logical parameters for local fields and couplers should also be considered. In the parameter setting problem^30^, we assign each node and each edge in the minor embedding graph such that: (1) for each node in the tree *T*_*i*_ expanded by the same vertex *i*, its value ${h^{\prime} }_{{i}_{k}}$ satisfies $\sum \,{h^{\prime} }_{{i}_{k}}={h}_{i}$, (2) for each edge in the tree *T*_*i*_ expanded by the same vertex *i*, the value ${J}_{{i}_{k},{i}_{k^{\prime} }}$ needs to be large enough to make sure all physical qubits that correspond to the same logical qubit to be of the same value and (3) for each edge in the minor embedding graph which is in the original graph, we could use the same *J*_*ij*_ value.

Our programmed implementation of these methods were written in C/C++ using the XACC programming framework^31^. XACC enables integration of the D-Wave solver application programming interface (SAPI) using a directive-based programming model. Pre-processing of the input *N* generated the Ising parameters for a logical Hamiltonian that was then embedded into the hardware graph structure. For minor embedding, we use the sapi_findembedding method included in the D-Wave 2000Q control software, SAPI version 3.0. This embedding methods is based on a randomized algorithm from Cai, Macready and Roy^32^, while access to these methods were managed using the XACC dwsapi-embedding plugin^31^. The corresponding biases and couplings for the embedded problem were generated using the logical Ising parameters. The output of the embedding was a program implementation of the physical Ising model that was submitted for execution on the D-Wave processor. Additional parameters for the execution included the number of samples *S* and the annealing duration *T*. The default annealing schedule for the 2000Q was used for all executions. The output from each of the *S* executions was a measured binary string designating ±1 values for each spin variable. The number of samples was *S* = 10,000. Each returned string was then classified according to the corresponding energy for the physical Ising model and subsequently decoded into the factors *p* and *q*. A histogram of all solutions returned for a specific annealing time was recorded.

**Figure 1:**
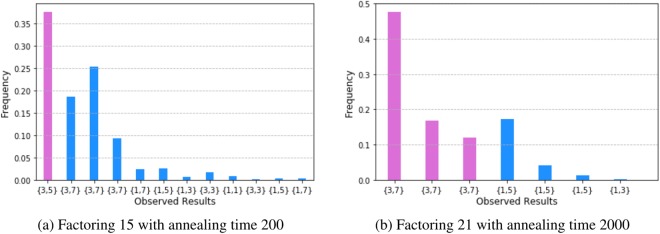
Experimental results on D-Wave machine: rates of getting different solutions. For example, the (3, 5) in the x-axis denotes the factorization of 15 is 3 multiplied by 5, the number in y-axis denotes the rate to get this factorization.

Figure 1(a,b) shows the frequency of each decoded solutions to the factorization problem for *N* = 15 and 21 using the direct method. These observed solutions are decoded using the inverse of the embedding with majority vote used to resolve any ambiguity in results. The plot presents the decoded results in order of lowest energy to highest energy (left to right). For these two examples, the lowest energy solution corresponds to the correct factors. In addition, several other computed solutions decoded into the correct factors as the associated errors were resolved by the decoding method. For example, several solutions are labeled as (3,7) because the observed bit strings corresponded to high-energy states before decoding. Only the first (leftmost) corresponds to the lowest energy state. The others were higher energy solutions, thus can’t be counted as the correct solution.

**Figure 2:**
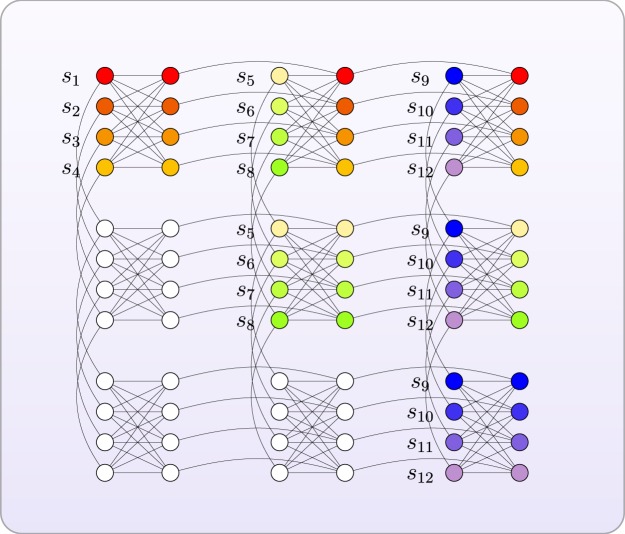
Embedding the factoring instance *N* = 143 to Chimera graph. The nodes with the same color denote the same original qubit, with their connected lines corresponding to strong couplings. The left footnotes refer to which spin the node was embedded.

Using the modified multiplication table method for factoring 143, we embed the problem Hamiltonian to D-Wave machine using the following method. Suppose *n* qubits are needed in the Hamiltonian, we divide *n* into $\lceil \frac{n}{4}\rceil$ groups. For each group, we use 4 copies of the nodes with each ${h^{\prime} }_{{i}_{k}}=\frac{1}{4}{h}_{i}$. We assign each edge in the tree *T*_*i*_ the negative number with largest absolute value to make it a penalty term. This method guarantees the nodes correspond to the same original qubit have the same value. We assign each edge corresponding to the original edge in the problem graph the same *J*_*ij*_ value. The embedded graph to D-Wave machine is in Fig. 2.

**Figure 3:**
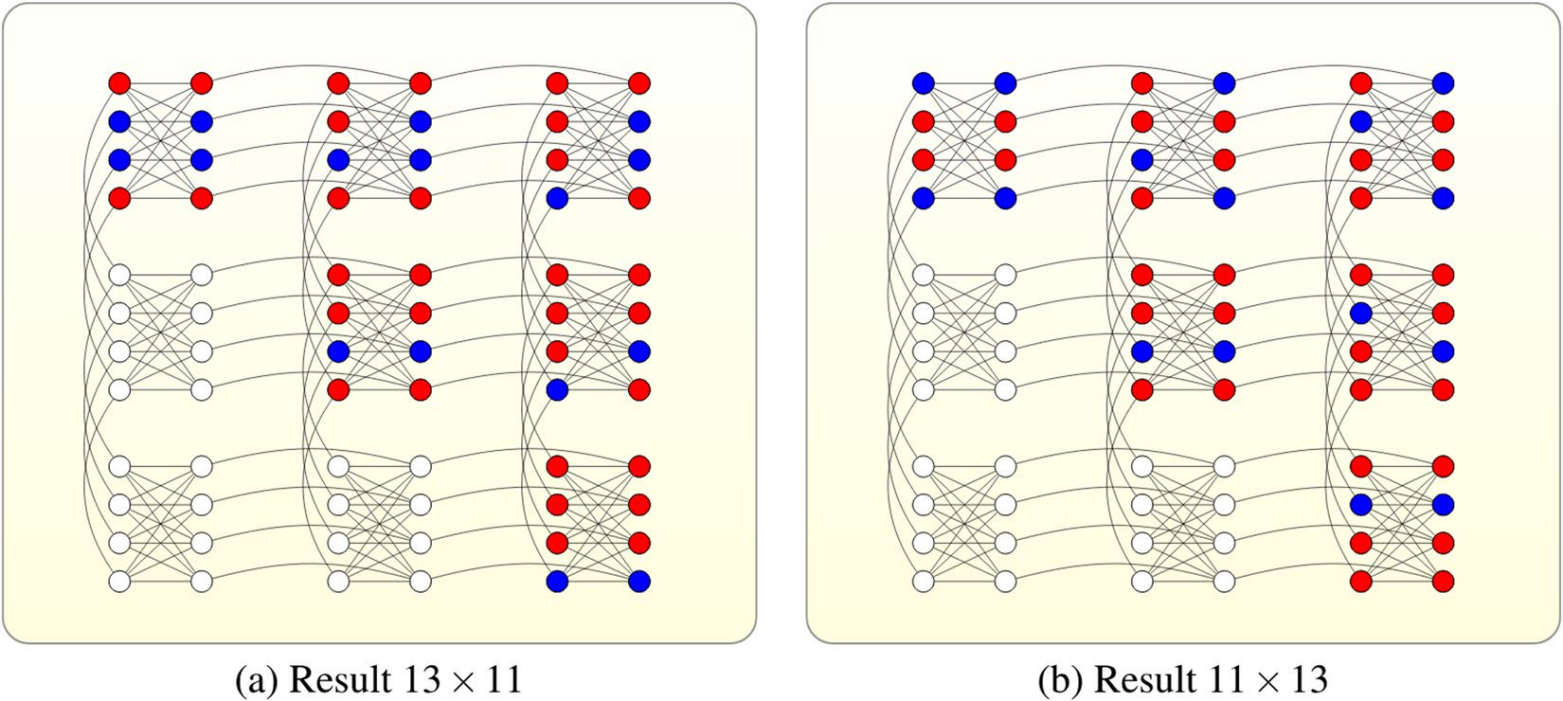
Experimental results on D-Wave machine: final ground state of factoring 143. Nodes colored red denote +1, nodes colored blue denote −1. (**a**) This graph shows *s*_1_ = 1, *s*_2_ = −1, *s*_3_ = −1, *s*_4_ = 1 which means *p* = 1101, *q* = 1011. (**b**) This graph shows *s*_1_ = −1, *s*_2_ = 1, *s*_3_ = 1, *s*_4_ = −1 which means *p* = 1011, *q* = 1101.

The results graph are shown in Fig. 3. The final state of the part of the system which represents the problem solution will be |1 −1 −1 1〉 or |−1 1 1−1〉 with relatively high probability, which corresponds to solutions *p* = (1*p*_2_*p*_1_1) = (1101)_2_ = 13, *q* = (1*q*_2_*q*_1_1) = (1011)_2_ = 11 or *p* = 11, *q* = 13.

For factoring larger numbers like 376289 using D-Wave, we embed the Hamiltonians into the Chimera hardware using the predefined function *find_embedding* and *embed_problem* available in the vendor’s software developer packages, because the problem graph can’t be embedded directly like the case 143 shown above which has 12 qubits, due to the size limitation of the current Chimera hardware. For now, the largest number experimented for finding an embedding in D-Wave 2000Q is 249919 which equals to 509 × 491. It uses 74 qubits in the final Ising Hamiltonian, and embeds to 1803 physical qubits in Chimera graph.

## Conclusions

We have presented two general methods for factoring integers using quantum annealing for optimizing a cost function that is reduced to an Ising Hamiltonian. Both methods requires ${\mathscr{O}}({\mathrm{log}}^{2}(N))$ qubits in total, where *N* is the number to be factored. The novelty of our demonstration of quantum annealing for prime factorization is based on the reduction in quantum resources required to execute factoring and the experimental verification of the algorithmic accuracy using currently available hardware. As a proof-of-concept, we have demonstrated these methods by factoring integers using the D-Wave 2000Q quantum annealing hardware, but these methods may be used on any other quantum annealing system with a similar number of qubits, qubit degree of connectivity, and hardware parameter precision. Assuming that quantum annealing hardware systems will continue to grow both in the number of qubits and bits of precision capabilities, our methods offer a promising path toward factor much larger numbers in the future. It is also good to combine our method with other ad hoc methods to achieve significantly better performances for specific numbers.

Finally, we note that while our demonstrations of factoring have made use of currently available quantum annealers, there is an outstanding question regarding the asymptotic complexity for this approach. It is well known that algorithmic complexity within the AQC model depends on the minimum spectral gap between the ground and first-excited states of the underlying time-dependent Hamiltonian. Attempts to classify the complexity of the spectral gap with respect to system size have not yet succeed and, indeed, Cubitt, Perez-Garcia, and Wolf have proven that the problem of claiming a Hamiltonian has a gap is undecidable in general^33^. Nonetheless, there is hope that our resource-efficient algorithms may find use in pre-processing potential factors for noisy factorization algorithms, e.g., as suggested by Patterson *et al*. within the context of RSA^34^.

## Electronic supplementary material

Supplemental material

## Data Availability

The data that support the plots within this paper and other findings of this study are available from the corresponding author upon reasonable request.
