# Supplementary material for: Quantum Annealing for Prime Factorization
Source: Sci Rep. 2018 Dec 5;8:17667. doi: 10.1038/s41598-018-36058-z (PMC6281593; doi:10.1038/s41598-018-36058-z)
Supplement: Supplementary file 1 — Supplemental material [file 41598_2018_36058_MOESM1_ESM.pdf]

Supplementary material for "Quantum Annealing for Prime Factorization"

Shuxian Jiang[1], Keith A. Britt[2], Alexander J. McCaskey[2], Travis S. Humble[*2], and Sabre Kais[†1,3]

[1]Department of Computer Science, Purdue University, West Lafayette, IN 47906
[2]Quantum Computing Institute, Oak Ridge National Laboratory, Oak Ridge, TN 37831
[3]Department of Chemistry, Physics and Birck Nanotechnology Center, Purdue University, West Lafayette, IN 47906

Factoring $N = 15 = 5 \times 3$

Define $p = (x_1 1)_2 = x_1 * 2 + 1, q = (x_2 x_3 1)_2 = x_2 * 2^2 + x_3 * 2 + 1, x_i \in \{0, 1\})$, p and q are prime numbers. The cost function is

$$\begin{aligned} & f(x_1, x_2, x_3) \\ = \; & (N - pq)^2 \\ = \; & [15 - (x_1 * 2 + 1)(x_3 * 2^2 + x_2 * 2 + 1)]^2 \\ = \; & 128x_1x_2x_3 - 56x_1x_2 - 48x_1x_3 + 16x_2x_3 - 52x_1 - 52x_2 - 96x_3 + 196. \end{aligned}$$

Use the replacement in Eq.1, we got

$$\begin{aligned} & f'(x_1, x_2, x_3, x_4) \\ = \; & 128(x_4x_3 + 2(x_1x_2 - 2x_1x_4 - 2x_2x_4 + 3x_4)) - 56x_1x_2 - 48x_1x_3 + 16x_2x_3 \\ & -52x_1 - 52x_2 - 96x_3 + 196 \\ = \; & 200x_1x_2 - 48x_1x_3 - 512x_1x_4 + 16x_2x_3 - 512x_2x_4 + 128x_3x_4 \\ & -52x_1 - 52x_2 - 96x_3 + 768x_4 + 196. \end{aligned}$$

with

$$\min_{x_1x_2=x_4} f(x_1, x_2, x_3, x_4) = \min f'(x_1, x_2, x_3, x_4)$$

because the coefficient of $x_1x_2x_3$ term is positive.

Then we do variable replacement using $x_i = \frac{1-s_i}{2}, i = 1, 2, 3, 4$

$$\begin{aligned} & f'(x_1, x_2, x_3, x_4) \\ = \; & 200\frac{1-s_1}{2}\frac{1-s_2}{2} - 48\frac{1-s_1}{2}\frac{1-s_3}{2} - 512\frac{1-s_1}{2}\frac{1-s_4}{2} + 16\frac{1-s_2}{2}\frac{1-s_3}{2} \\ & -512\frac{1-s_2}{2}\frac{1-s_4}{2} + 128\frac{1-s_3}{2}\frac{1-s_4}{2} - 52\frac{1-s_1}{2} - 52\frac{1-s_2}{2} \\ & -96\frac{1-s_3}{2} + 768\frac{1-s_4}{2} + 196 \\ = \; & 116s_1 + 100s_2 + 24s_3 - 160s_4 + 50s_1s_2 - 12s_1s_3 - 128s_1s_4 + 4s_2s_3 - 128s_2s_4 + 32s_3s_4 + 298 \\ = \; & 2g(s_1, s_2, s_3, s_4) \end{aligned}$$

*humblets@ornl.gov
†kais@purdue.edu

$g(s_1,s_2,s_3,s_4)$ is the energy function of

$$\begin{aligned}
& H_P(\sigma_z^{(1)},\sigma_z^{(2)},\sigma_z^{(3)},\sigma_z^{(4)}) \\
= \; & 58\sigma_z^{(1)}+50\sigma_z^{(2)}+12\sigma_z^{(3)}-80\sigma_z^{(4)}+25\sigma_z^{(1)}\sigma_z^{(2)}-6\sigma_z^{(1)}\sigma_z^{(3)}-64\sigma_z^{(1)}\sigma_z^{(4)}+2\sigma_z^{(2)}\sigma_z^{(3)}-64\sigma_z^{(2)}\sigma_z^{(4)} \\
& +16\sigma_z^{(3)}\sigma_z^{(4)}+149I.
\end{aligned}$$

Factoring $N = 143 = 13 \times 11$

We get the following equations for each blocks from multiplication table for factoring 143.

$$\begin{aligned}
(p_2+p_1q_1+q_2-(c_2\times 4+c_1\times 2))\times 2+(p_1+q_1) &= (11)_2=3 \\
(q_1+p_2q_2+p_1+c_2-(c_4\times 4+c_3\times 2))\times 2+(1+p_2q_1+p_1q_2+1+c_1) &= (01)_2=1 \\
(1+c_4)\times 2+(q_2+p_2+c_3) &= (100)_2=4
\end{aligned}$$

They could be further simplified as

$$\begin{aligned}
2p_2+2p_1q_1+2q_2-8c_2-4c_1+p_1+q_1-3 &= 0 \\
2q_1+2p_2q_2+2p_1+2c_2-8c_4-4c_3+p_2q_1+p_1q_2+c_1+1 &= 0 \\
q_2+p_2+c_3+2c_4-2 &= 0
\end{aligned}$$

We define the cost function to be squares of the left of equations. That is

$$\begin{aligned}
& f(p_1,p_2,q_1,q_2,c_1,c_2,c_3,c_4) \\
= \; & (2p_2+2p_1q_1+2q_2-8c_2-4c_1+p_1+q_1-3)^2+(2q_1+2p_2q_2+2p_1+2c_2-8c_4-4c_3+p_2q_1+p_1q_2+c_1+1)^2 \\
& +(q_2+p_2+c_3+2c_4-2)^2
\end{aligned}$$

Expand and simplify the function using the property $x^2=x$ for $x=0,1$. Then reduce the higher order terms to two order terms according to the following rule noticing that there will be negative high order terms:

$$\begin{cases} x_1x_2x_3 = x_4x_3+2(x_1x_2-2x_1x_4-2x_2x_4+3x_4) & \text{if } x_4=x_1x_2 \\ x_1x_2x_3 < x_4x_3+2(x_1x_2-2x_1x_4-2x_2x_4+3x_4) & \text{if } x_4\neq x_1x_2 \end{cases}$$

$$and \begin{cases} -x_1x_2x_3 = -x_4x_3+2(x_1x_2-2x_1x_4-2x_2x_4+3x_4) & \text{if } x_4=x_1x_2 \\ -x_1x_2x_3 < -x_4x_3+2(x_1x_2-2x_1x_4-2x_2x_4+3x_4) & \text{if } x_4\neq x_1x_2 \end{cases}$$

So the negative term $-x_1x_2x_3$ could be transformed to quadratic term in the same way as the positive term $x_1x_2x_3$.

The cost function could be minimized as long as the transformed one is minimized

$$\min(x_1x_2x_3) = \min(x_4x_3+2(x_1x_2-2x_1x_4-2x_2x_4+3x_4) \quad (1)$$

$$\min(-x_1x_2x_3) = \min(-x_4x_3+2(x_1x_2-2x_1x_4-2x_2x_4+3x_4) \quad (2)$$

Replace p_1q_1 with t_1,p_1q_2 with t_2, p_2q_2 with t_3, p_2q_1 with t_4, using the variable replacement rule if the coefficient of the term is positive or negative respectively. The cost function becomes

$$\begin{aligned}
& f(p_1,p_2,q_1,q_2,c_1,c_2,c_3,c_4,t_1,t_2,t_3,t_4) \\
= \; & 43c_1+120c_2+5c_3+44c_4+3p_1-11p_2+3q_1-11q_2+444t_1+252t_2+372t_3+252t_4+68c_1c_2-8c_1c_3 \\
& -16c_1c_4-16c_2c_3-32c_2c_4+68c_3c_4-4c_1p_1-16c_1p_2-8c_2p_1-32c_2p_2-16c_3p_1+2c_3p_2-32c_4p_1+4c_4p_2 \\
& -4c_1q_1-16c_1q_2-8c_2q_1-32c_2q_2-16c_3q_1+2c_3q_2-32c_4q_1+4c_4q_2-16c_1t_1+2c_1t_2-32c_2t_1+4c_1t_3 \\
& +4c_2t_2+2c_1t_4+8c_2t_3-8c_3t_2+4c_2t_4-16c_3t_3-16c_4t_2-8c_3t_4-32c_4t_3-16c_4t_4+4p_1p_2+158p_1q_1 \\
& +95p_1q_2+95p_2q_1+142p_2q_2+4q_1q_2-296p_1t_1-168p_1t_2+12p_2t_1+12p_2t_2-248p_2t_3-168p_2t_4-296q_1t_1 \\
& +12q_2t_1-168q_2t_2-168q_1t_4-248q_2t_3+12q_2t_4+2t_1t_3+14
\end{aligned}$$

$$
\begin{array}{cccccccccccc}
p_1 & p_2 & q_1 & q_2 & c_1 & c_2 & c_3 & c_4 & t_1 & t_2 & t_3 & t_4 \\
\downarrow & \downarrow & \downarrow & \downarrow & \downarrow & \downarrow & \downarrow & \downarrow & \downarrow & \downarrow & \downarrow & \downarrow \\
s_1 & s_2 & s_3 & s_4 & s_5 & s_6 & s_7 & s_8 & s_9 & s_{10} & s_{11} & s_{12}
\end{array}
$$

Then we do a variable transformation to make the variable in the domain {-1,1} using $x_i = \frac{1-s_i}{2}$ if we let $x_1 = p_1, x_2 = p_2, ..., x_{12} = t_4$.

$$
\begin{aligned}
& f'(p_1, p_2, q_1, q_2, c_1, c_2, c_3, c_4, t_1, t_2, t_3, t_4) \\
= \; & 2f(p_1, p_2, q_1, q_2, c_1, c_2, c_3, c_4, t_1, t_2, t_3, t_4) \\
= \; & (261s_1)/2 + (215s_2)/2 + (261s_3)/2 + (215s_4)/2 - 41s_5 - 82s_6 + 3s_7 + 6s_8 - 137s_9 - 81s_{10} - 107s_{11} - 81s_{12} \\
& +2s_1s_2 + 79s_1s_3 + (95s_1s_4)/2 + (95s_2s_3)/2 - 2s_1s_5 + 71s_2s_4 - 4s_1s_6 - 8s_2s_5 + 2s_3s_4 - 8s_1s_7 - 16s_2s_6 \\
& -2s_3s_5 - 16s_1s_8 + s_2s_7 - 4s_3s_6 - 8s_4s_5 - 148s_1s_9 + 2s_2s_8 - 8s_3s_7 - 16s_4s_6 - 84s_1s_{10} + 6s_2s_9 - 16s_3s_8 \\
& +s_4s_7 + 34s_5s_6 + 6s_2s_{10} - 148s_3s_9 + 2s_4s_8 - 4s_5s_7 - 124s_2s_{11} + 6s_4s_9 - 8s_5s_8 - 8s_6s_7 - 84s_2s_{12} - 84s_4s_{10} \\
& -8s_5s_9 - 16s_6s_8 - 84s_3s_{12} - 124s_4s_{11} + s_5s_{10} - 16s_6s_9 + 34s_7s_8 + 6s_4s_{12} + 2s_5s_{11} + 2s_6s_{10} + s_5s_{12} + 4s_6s_{11} \\
& -4s_7s_{10} + 2s_6s_{12} - 8s_7s_{11} - 8s_8s_{10} - 4s_7s_{12} - 16s_8s_{11} - 8s_8s_{12} + s_9s_{11} + 808
\end{aligned}
$$

This corresponds to Ising Hamiltonian with local fields

$$
\mathbf{h}^T = \begin{array}{c} \\ \end{array}
\begin{array}{cccccccccccc}
\sigma_z^{(1)} & \sigma_z^{(2)} & \sigma_z^{(3)} & \sigma_z^{(4)} & \sigma_z^{(5)} & \sigma_z^{(6)} & \sigma_z^{(7)} & \sigma_z^{(8)} & \sigma_z^{(9)} & \sigma_z^{(10)} & \sigma_z^{(11)} & \sigma_z^{(12)} \\
130.5 & 107.5 & 130.5 & 107.5 & -41 & -82 & 3 & 6 & -137 & -81 & -107 & -81
\end{array}
$$

and coupling terms:

$\mathbf{J}$ =

	$\sigma_z^{(1)}$	$\sigma_z^{(2)}$	$\sigma_z^{(3)}$	$\sigma_z^{(4)}$	$\sigma_z^{(5)}$	$\sigma_z^{(6)}$	$\sigma_z^{(7)}$	$\sigma_z^{(8)}$	$\sigma_z^{(9)}$	$\sigma_z^{(10)}$	$\sigma_z^{(11)}$	$\sigma_z^{(12)}$
$\sigma_z^{(1)}$		2	79	47.5	−2	−4	−8	−16	−148	−84	0	0
$\sigma_z^{(2)}$			47.5	71	−8	−16	1	2	6	6	−124	−84
$\sigma_z^{(3)}$				2	−2	−4	−8	−16	−148	0	0	−84
$\sigma_z^{(4)}$					−8	−16	1	2	6	−84	−124	6
$\sigma_z^{(5)}$						34	−4	−8	−8	1	2	1
$\sigma_z^{(6)}$							−8	−16	−16	2	4	2
$\sigma_z^{(7)}$								34	0	−4	−8	−4
$\sigma_z^{(8)}$									0	−8	−16	−8
$\sigma_z^{(9)}$										0	1	0
$\sigma_z^{(10)}$											0	0
$\sigma_z^{(11)}$												0
$\sigma_z^{(12)}$												

Range of Coefficients

Define the lengths of p and q as l_1 and l_2, respectively. Let $l_1 = \frac{log(N)}{2} = O(log(N))$ and $l_2 = \frac{log(N)}{2} = O(log(N))$. Suppose each block contains 3 columns as in Table 2 for factoring 59989. Then the sum for one block is not larger than $(l_2+1)+2(l_2+1)+4(l_2+1) = 7l_2+7$, assuming all unknown bits in p and q are 1's and all carries from the block on the right hand side are 1's. Thus, the length of the sum for the current block is at most $log(7l_2+7) = O(log(log(N)))$. Therefore, the length of the carry from the sum of current block is at most $log(7l_2+7)-3 = O(log(log(N)))$. Because the length of the carry plus the width of the block (which is 3 in this case) determines the range of the coefficients

in the cost function, the maximum coefficient in the cost function corresponding to one block is $(2^{O(log(log(N)))})^2 = O((log(N))^2)$. This square comes from transforming the equation for each block to a square that makes the equation hold. (See Sec.2 for examples of these equations.) There are approximately $\frac{log(N)}{3}$ blocks in total for this example, such that the coefficient in the combined cost function containing all cost functions for each block is no larger than $\frac{log(N)}{3} * O((log(N))^2) = O((log(N))^3)$. Note that for the majority of cases, this becomes $O((log(N))^2)$ because most of terms in different blocks are different. Since the variable replacement only effects the scale of the range of the coefficients linearly, the coefficients of the final quadratic cost function are polynomially large with regard to the size of N, the number to be factored.
